# Prolonged viral shedding of SARS-CoV-2 and related factors in symptomatic COVID-19 patients: a prospective study

**DOI:** 10.1186/s12879-021-07002-w

**Published:** 2021-12-27

**Authors:** Hui Long, Jing Zhao, Hao-Long Zeng, Qing-Bin Lu, Li-Qun Fang, Qiang Wang, Qing-Ming Wu, Wei Liu

**Affiliations:** 1 Tianyou Hospital, Wuhan University of Science and Technology, Wuhan, Hubei People’s Republic of China; 2 State Key Laboratory of Pathogen and Biosecurity, Beijing Institute of Microbiology and Epidemiology, Beijing, People’s Republic of China; 3 Department of Laboratory Medicine, Tongji Hospital, Tongji Medical College, Huazhong University of Science and Technology, Wuhan, People’s Republic of China; 4 Department of Laboratorial Science and Technology, School of Public Health, Peking University, Beijing, People’s Republic of China; 5 Institute of Infection, Immunology and Tumor Microenvironment, Hubei Province Key Laboratory of Occupational Hazard Identification and Control, Medical College, Wuhan University of Science and Technology, Wuhan, People’s Republic of China

**Keywords:** COVID-19, SARS-CoV-2, Viral shedding, Antibody, Risk factor

## Abstract

**Background:**

The temporal relationship between SARS-CoV-2 and antibody production and clinical progression remained obscure. The aim of this study was to describe the viral kinetics of symptomatic patients with SARS-CoV-2 infection and identify factors that might contribute to prolonged viral shedding.

**Methods:**

Symptomatic COVID-19 patients were enrolled in two hospitals in Wuhan, China, from whom the respiratory samples were collected and measured for viral loads consecutively by reverse transcriptase quantitative PCR (RT-qPCR) assay. The viral shedding pattern was delineated in relate to the epidemiologic and clinical information.

**Results:**

Totally 2726 respiratory samples collected from 703 patients were quantified. The SARS-CoV-2 viral loads were at the highest level during the initial stage after symptom onset, which subsequently declined with time. The median time to SARS-CoV-2 negativity of nasopharyngeal test was 28 days, significantly longer in patients with older age (> 60 years old), female gender and those having longer interval from symptom onset to hospital admission (> 10 days). The multivariate Cox regression model revealed significant effect from older age (HR 0.73, 95% CI 0.55–0.96), female gender (HR 0.72, 95% CI 0.55–0.96) and longer interval from symptom onset to admission (HR 0.44, 95% CI 0.33–0.59) on longer time to SARS-CoV-2 negativity. The IgM antibody titer was significantly higher in the low viral loads group at 41–60 days after symptom onset. At the population level, the average viral loads were higher in early than in late outbreak periods.

**Conclusions:**

The prolonged viral shedding of SARS-CoV-2 was observed in COVID-19 patients, particularly in older, female and those with longer interval from symptom onset to admission.

**Supplementary Information:**

The online version contains supplementary material available at 10.1186/s12879-021-07002-w.

## Background

COVID-19 pandemic caused by severe acute respiratory syndrome coronavirus 2 (SARS-CoV-2) has affected over 166.9 million patients with more than 3.5 million deaths all over the world until May 24, 2021 [1]. Despite of stringent control measures, COVID-19 continues to circulate worldwide, severely disrupted the health care system and halted socioeconomic activities. The confirmation of SARS-CoV-2 infection in clinical practice relies on the detection of virus RNA in various types of samples during the acute phase of infection. The prolonged shedding of virus RNA in various body fluids beyond the acute phase of infection has been reported in various studies [2–5], which is auxiliary in making the clinical diagnosis and preventing of onward virus transmission. COVID-19 encompasses a heterogeneous spectrum of illness, ranging from asymptomatic and mild infections, to severely ill cases in 4–16% [6, 7]. How were the duration and magnitude of SARS-CoV-2 RNA can be related to clinical phenotypes had been investigated in the early phase of the epidemic and commonly in small-scale studies, which results lacked replication by large scale study [8, 9]. As the disease progressed, whether this association remained unchanged remained to be explored. Viral replication and overwhelming immune responses are believed to contribute to the clinical outcome of COVID-19, however, the temporal relationship between viral load and antibody production and clinical progression remained obscure. Here we performed a hospital-based study on two cohorts of confirmed symptomatic COVID-19 patients to acquire a detailed understanding of the dynamics of SARS-CoV-2 infection, to record the time that detection of SARS-CoV-2 turned to negative for all the patients and to estimate the factors.

## Methods

### Patient enrollment

From January 2020 to March 2020, COVID-19 patients were recruited from two COVID-19 referral hospitals in Wuhan, Hubei Province, i.e., Tongji hospital and Tianyou hospital. The COVID-19 was diagnosed based on the standard protocol released by the National Health Commission of China. Four types of detection kits (Da An Gene, BioGerm, TIANLONG Technology and HUIRUI Co., Ltd) were used to test for SARS-CoV-2 RNA in the collected nasopharyngeal swab (NPS) specimens following the World Health Organization (WHO) guidelines (World Health Organization, 2020) [10–12]. For the confirmed COVID-19 patients, sequential NPS samples were collected for SARS-CoV-2 RNA quantification by the cycle threshold value (Ct value) during their hospitalization until discharge or death. The lower detection limit of assay was 1 × 10^3^ copies/mL. Totally 45 cycles have been run for RT-qPCR test, and samples with Ct value < 40 were considered as positive, while Ct value ≥ 40 and undetectable Ct value were considered as negative. Two target genes, the nucleocapsid protein (N) and open reading frame 1ab (ORF1ab) genes were simultaneously amplified, and the samples positive for either target were considered as positive. Across the study, the same protocols and reagents were used for the tests. Time to SARS-CoV-2 RNA negativity was defined as the days of interval between the date of disease onset and the date of SARS-CoV-2 RNA turning negative.

The serum samples collected at the diagnosis and before discharge were used for the measurement of SARS-CoV-2-specific IgM and IgG antibodies by using a seroFlash SARS-CoV-2 IgG/IgM ELISA fast kit (Epigentek, USA) with a recombinant SARS-CoV-2 antigen (Spike protein).

### Data collection

Medical chart review was conducted for the recruited patients to collect information regarding demographics, medical history, disease onset (the 1st day of reporting symptoms consistent with COVID-19) and clinical phenotypes. The severity of illness was defined into severe or mild disease according to the 7th edition of Chinese Clinical Guidance for COVID-19 Pneumonia Diagnosis and Treatment issued by the National Health Commission of the People’s Republic of China by a group of trained medical staff [12].

### Statistical analyses

Continuous variables were expressed as median and interquartile range (IQR) or mean ± standard deviation (SD) and categorical variables were expressed as frequencies. Fisher exact test, Wilcoxon rank sum test, or log rank test was used for inter-group comparisons and logistic regression model for multivariable analysis. Chi-square test or Wilcoxon rank sum test was performed depending on parametric or nonparametric data. Survival analysis was performed using Kaplan–Meier curves and Cox regression model to identify potential factors that were related to the time to SARS-CoV-2 RNA negativity. All statistical analyses were performed using the R software (version 3.5.3). A two-sided P value < 0.05 was considered statistically significant.

## Results

### Patient recruit and baseline information

A total of 2726 NPS samples from 703 enrolled patients were quantified for SARS-CoV-2 RNA (median of 3 samples per patient). The median age of the study patients was 63 years (range 10–92 years), and 332 (47.2%) were male. The median interval from symptom onset to the hospital admission was 11 days (IQR 7–15). At least one underlying medical condition was recorded from 290 (41.3%) patients, comprised of hypertension in 236 (33.6%), diabetes in 99 (14.1%), cardiovascular disease in 53 (7.5%) and cerebral infarction in 19 (2.7%) of the patients. Most of the patients (87.6%, 616/703) had mild disease and the remaining 12.4% had severe disease. The commonly seen clinical symptoms, including fever, cough, fatigue, anhelation, nausea, diarrhea and anorexia, were observed with comparable frequencies between mild and severe patients (all P > 0.05, Table 1).

**Table 1:** Demographics and clinical characteristics of recruited COVID-19 patients

Characteristics	Total	Mild	Severe	P value
	(n = 703)	(n = 616)	(n = 87)	
Age, years, median (IQR)	63 (50–70)	62 (49–69)	67 (57–76)	< 0.001
≤ 60	307 (43.7)	281 (45.6)	26 (29.9)	0.006^*^
> 60	396 (56.3)	335 (54.4)	61 (70.1)	
Sex, male, n (%)	332 (47.2)	281 (45.6)	51 (58.6)	0.023^*^
Interval^#^, days, median (IQR)	11 (7–15)	11 (7–15)	10 (7–15)	0.808
≤ 10	348 (49.5)	303 (49.2)	45 (51.7)	0.658
> 10	355 (50.5)	313 (50.8)	42 (48.3)	
Length of stay, days, median (IQR)	21 (14–24)	22 (15–24)	18 (10–23)	0.001
Clinical manifestation on admission, n (%)				
Fever	533 (75.8)	467 (75.8)	66 (75.9)	0.992
Cough	438 (62.3)	387 (62.8)	51 (58.6)	0.449
Fatigue	174 (24.8)	149 (24.2)	25 (28.7)	0.358
Anhelation	178 (25.3)	149 (24.2)	29 (33.3)	0.066
Nausea	57 (8.1)	50 (8.1)	7 (8.0)	0.982
Diarrhea	115 (16.4)	104 (16.9)	11 (12.6)	0.317
Anorexia	72 (10.2)	65 (10.6)	7 (8.0)	0.471
Comorbidity, n (%)	290 (41.3)	245 (39.8)	45 (51.7)	0.034
Hypertension	236 (33.6)	199 (32.3)	37 (42.5)	0.059
Diabetes	99 (14.1)	83 (13.5)	16 (18.4)	0.217
Cardiovascular disease	53 (7.5)	47 (7.6)	6 (6.9)	0.808
Cerebral infarction	19 (2.7)	15 (2.4)	4 (4.6)	0.417

*IQR* interquartile range

*Significant difference between mild and severe patients after adjusting for age, sex and presence of comorbidity

^#^Indicates interval between disease onset and hospital admission

### Dynamic profile of viral load and detection rates of SARS-CoV-2

**Fig. 1:**
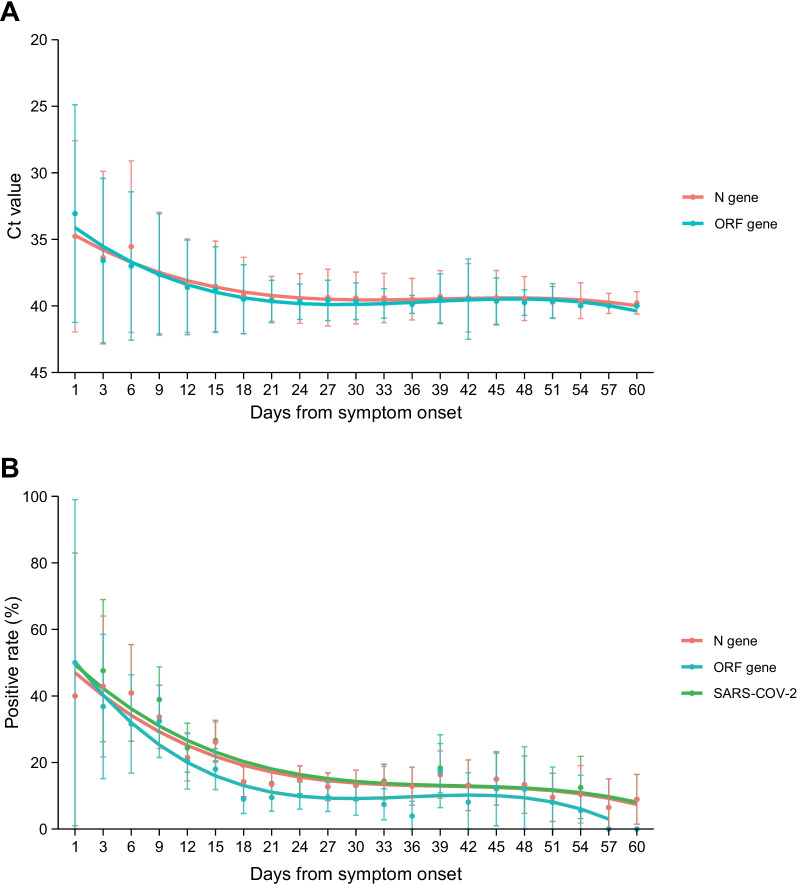
Dynamic profile of viral loads and positive rates of SARS-CoV-2 based on N and ORF genes amplification among COVID-19 patients during the whole process of hospitalization. **A** The dynamic profile of viral loads [cycle threshold (Ct) value] measured by RT-qPCR assay using N and ORF specific primers in COVID-19 patients; **B** the dynamic profile of positive rates measured by N and ORF specific primers in COVID-19 patients. The positive for SARS-CoV-2 was defined by either positive for N or ORF of SARS-CoV-2. When applicable, mean ± standard deviation (SD) of Ct value is shown. Dots and error bars denote means and SDs, respectively

The viral load and positive rates using both N and ORF specific primers showed declining trends (Fig. 1). The specimens collected in the initial stage of disease onset at the diagnosis had the highest positive rate (mean of 42.9% for N gene on the 3rd day of symptom onset and 50% for ORF gene on the 1st day of symptom onset), followed by consistently decrease thereafter, till to 5.9% for N gene and 0% for ORF at the last observation. In a consistent manner, the Ct value of N and ORF genes increased obviously from 34.76 ± 7.18 and 33.06 ± 8.18 (mean ± SD) at the diagnosis to 39.54 ± 1.71 and 39.64 ± 1.53 at 21st day after symptoms onset, respectively, followed by a plain level of Ct value of 40 that last for the remaining tests. According to the fitted temporal curves, both viral load and positive rates correlated negatively with time after onset of symptoms (R^2^ of Ct values were 0.96 and 0.92 for N and ORF genes; R^2^ of positive rates were 0.93 and 0.94 for N and ORF genes, all P < 0.001), and with a faster decay of positive rate for ORF than N gene (decay rate, 0.83% vs. 0.63%) (Fig. 1).

### The prolonged viral shedding and associated factors

**Fig. 2:**
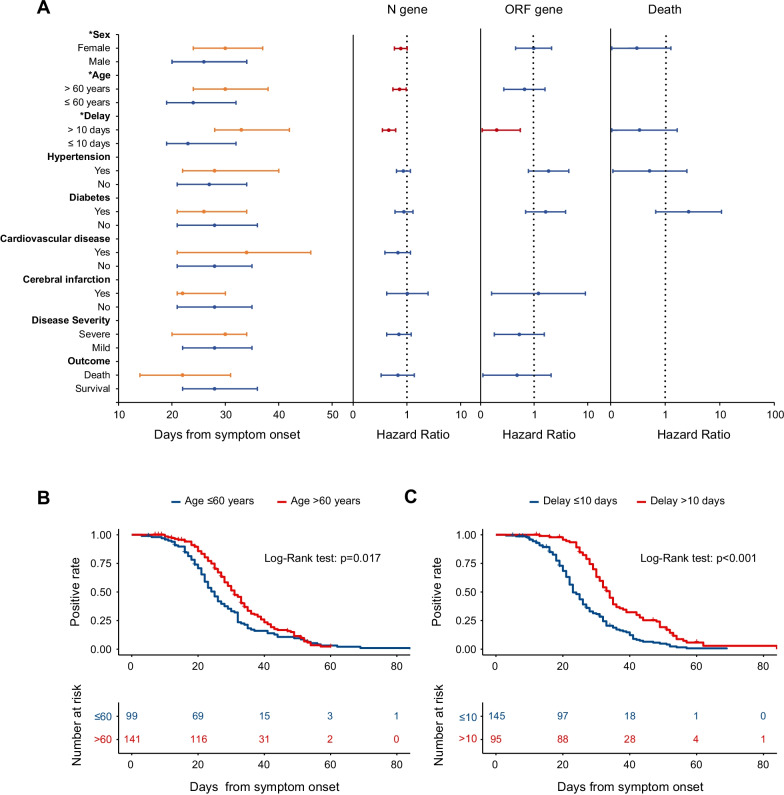
The time of SARS-CoV-2 RNA negativity in relation with demographic information among hospitalized COVID-19 patients. **A** The length of time to RT-PCR negativity compared between groups of patients based on Wilcoxon ranks sum test, asterisks represent significant differences between groups (P < 0.05). Dots and error bars denote medians and interquartile ranges, respectively (Left column). The hazard ratio (HR) and 95% confidence interval (CI) were displayed for the Cox regression model based on N, ORF gene and death outcome, the dots are the HRs and the error bars are the 95%CIs. The red color represents P < 0*.*05 and the blue color represents P ≥ 0*.*05. The dotted line indicates an OR of 1 (Middle and right column); **B** Kaplan–Meier curves compared by log-rank test on probability of RT-qPCR negativity according to age; **C** Kaplan–Meier curves compared by log-rank test on probability of RT-qPCR negativity according to the delay from symptom onset to hospital admission

Among 240 COVID-19 patients who had over three SARS-CoV-2 RNA tests, 214 patients had converted negative to the end of the observation. An overall CFR of 4.2% (9/214) was obtained. Inter-group comparisons showed median time to SARS-CoV-2 RNA negativity was significant longer in patients who were female, aged > 60 years and with longer interval (> 10 days) from symptoms onset to hospital admission (P = 0.034, P = 0.003 and P < 0.001, respectively, Fig. 2A). Based on N gene, 205 (85.4%) COVID-19 patients had converted negative to the end of the observation (Additional file 1: Table 1). The median time of SARS-CoV-2 RNA negativity was 28 days (IQR 22–36), 100 patients had viral shedding > 28 days (long viral shedding, LVS group), and 105 patients has viral shedding ≤ 28 days (short viral shedding, SVS group) in the NPS samples. Inter-group comparisons disclosed that the LVS group had significantly higher frequency of female (55% vs. 39%), older age (> 60 years, 66% vs. 49.5%), with longer interval from symptom onset to hospital admission (12 vs. 8 days), longer length of hospitalization (24 vs. 23 days) and higher frequency of cough (73% vs. 55.2%) than the SVS group (all P < 0.05). Based on the test of ORF gene, the median duration of viral shedding was 24 days (IQR 22–29), the long viral shedding > 24 days was significantly associated with longer interval from symptom onset to admission (11 vs*.* 8 days) than those with short viral shedding (P < 0.05, Additional file 1: Table 1). Higher frequency of severe disease was observed in the LVS group than in SVS group, which difference, however, was insignificant (21.4% vs*.* 7.1%, P = 0.596).

### Survival analysis based on time to SARS-CoV-2 RNA negativity

The univariate Cox regression model disclosed that patients with delayed negative conversion (> 28 days according to N gene test) was related to female, older age (> 60 years) and longer interval from symptom onset to admission (> 10 days) (all P < 0.05, Fig. 2A). Likewise, Kaplan–Meier curves revealed the same effect from older age (> 60 years), and interval > 10 days from symptom onset to admission on longer time to SARS-CoV-2 RNA negativity (P = 0.017 and P < 0.001, respectively, Fig. 2B and C). In a consistent manner, the multivariate Cox regression model analysis revealed that interval from symptom onset to admission > 10 days, female gender and older age > 60 years were related to late negative conversion (HR 0.44, 95% CI 0.33–0.59, HR 0.72, 95% CI 0.55–0.96 and HR 0.73, 95% CI 0.55–0.96, respectively, all P < 0.05, Table 2). No significant effect on time to SARS-CoV-2 negativity was observed from disease severity based on either univariate or multivariate Cox regression model (P > 0.05, Table 2). When the recorded clinical manifestations on admission were further evaluated, no association was observed between any symptom (fever, cough, fatigue, anhelation, nausea, diarrhea or anorexia) and prolonged viral shedding (Additional file 1: Table 2).

**Table 2:** The association between longer time to SARS-CoV-2 RNA negativity and age, sex, interval between disease onset and hospital admission, disease severity for COVID-19 patients

Characteristics	Crude		Adjusted*	
	HR (95% CI)	P value	HR (95% CI)	P value
Age, years				
≤ 60	Reference		Reference	
> 60	0.72 (0.55–0.95)	0.019	0.73 (0.55–0.96)	0.025
Sex				
Male	Reference		Reference	
Female	0.79 (0.60–1.03)	0.083	0.72 (0.55–0.96)	0.023
Interval^#^, days				
≤ 10	Reference		Reference	
> 10	0.45 (0.34–0.60)	< 0.001	0.44 (0.33–0.59)	< 0.001
Disease severity				
Mild	Reference		Reference	
Severe	0.72 (0.44–1.21)	0.217	0.72 (0.42–1.21)	0.216

*HR* hazard ratio; *CI* confidence interval

*Independent variables including age, sex and any comorbidity were included into multivariate Cox regression model

^#^Indicates interval between disease onset and hospital admission. The univariate and multivariate Cox regression model were used in the analysis

### Anti-SARS-CoV-2 IgM and IgG antibody in relation to viral load

Considering that IgM and IgG antibodies were only detectable around 20th day after symptom onset, we divided the clinical progression into two stages (21st–40th day and 41st–60th day post disease onset), and within each stage, the correlation between antibody levels and SARS-CoV-2 viral load were separately investigated. Based on the minimum Ct value per patient, high viral loads that exceeded mean Ct (herein Ct of 36.3 for N gene and Ct of 37.9 for ORF gene) and low viral loads that below mean Ct were grouped into the HVL and LVL group, separately.

The geometric mean reciprocal titer (GMRT) of the antibodies evaluated during two stages were separately compared between HVL vs. LVL groups, and only significantly higher IgM antibody titer was found in the LVL group based on the N gene evaluation at the second stage (Additional file 1: Fig. 1A and B). No significant difference in IgG antibody titer was observed between LVL and HVL groups during either stages (Additional file 1: Fig. 1C and D).

### Outbreak kinetics of viral loads level

**Fig. 3:**
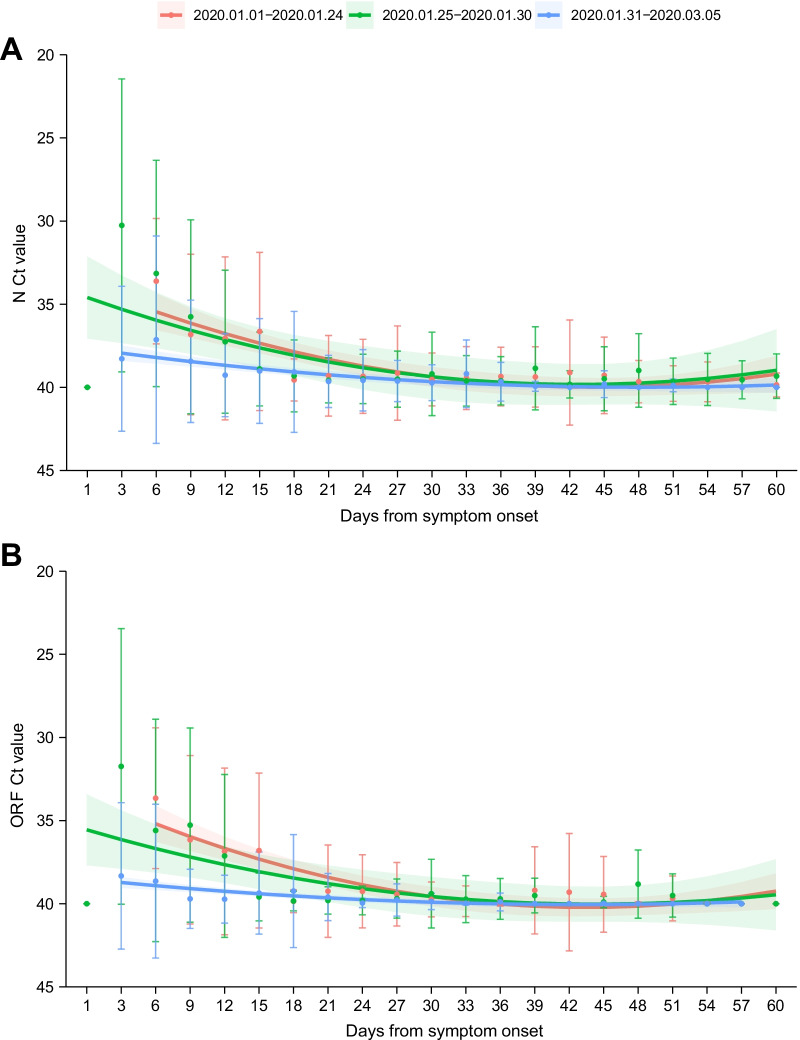
Dynamic profile on viral loads detected by N gene (**A**) and ORF gene (**B**) among COVID-19 patients across the outbreak periods. Three periods (early, middle and late) were approximate equally classified based on the date of symptom onset of the included patients: 1st–24th January 2020 (red line, 229 patients), 25th–30th January 2020 (green line, 245 patients), and 31st January–5th March 2020 (blue line, 228 patients). Dots and error bars denote means and SDs, respectively

We sought to determine if the SARS-CoV-2 viral loads of patients upon admission to the hospital changed over the course of the outbreak, regardless of outcome. Based on the symptom onset date of the patients, three outbreak stages (early, middle and late) were classified to attain comparable case numbers among three stages, i.e., with disease onset from 1st to 24th January 2020 (red line, 229 patients), 25th to 30th January 2020 (green line 245 patients), and 31st January to 5th March 2020 (blue line, 228 patients). The viral load initially evaluated before therapy from each patient were plotted over the course of three periods and fitted to a polynomial regression. GEE models based on either N or ORF gene, demonstrated significantly lower viral loads from the patients hospitalized at the third stage when compared with those hospitalized at early epidemic after adjusting for the potential effect from age, sex, days from symptom onset to admission, and disease severity (OR 1.53, 95% CI 1.07–2.20 for N gene; OR 1.94, 95% CI 1.32–2.87 for ORF gene; both P < 0.05, Additional file 1: Table 3 and Fig. 3).

## Discussion

In the current cohort study, the temporal pattern of viral shedding from NPS were determined, and compared in relate to their demographic features and the clinical severity. We found the peak viral load occurred early for both genes, similar to He’s studies [13], which decreased gradually after onset of symptoms, as patient’s immune system responded to infection. Moreover, moderate level of viral shedding was still present at 21st day after onset of symptoms, which decreased to positive rate of 0.7% among the patients tested for SARS-CoV-2 at the end of observation of more than 2 months post disease. We found that the median time to SARS-CoV-2 negativity in NPS was 26 days with 95th percentile of 53 days, thus detection of virus RNA in NPS samples at the 60th day after illness onset should be a low-probability event, beyond the 95th percentile limit.

Long viral shedding has been previously recorded, from 4 weeks in Zheng’s study and 20 days in To et al.’s study [8, 14]. Zheng et al. also demonstrated longer time until loss of virus RNA detection in severe cases than in mild cases [8]. Zhou et al. found prolonged presence of SARS-CoV-2 viral RNA in 137 samples as long as 37 days [15]. In contrast, Lan et al. and Lescure et al. reported positive rRT-PCR detection not exceeding 2 weeks, but only based on small sample size [5, 16]. Here based on a large sample size of symptomatic patients, we simulated a curve that was reflective of the dynamic profile of viral load, we can determine the viral RNA level that could be derived from a patient but only limited to patients with symptomatic infection, while not for those with asymptomatic infection.

The test based on two genes displayed different duration of SARS-CoV-2 RNA positive test, which was longer for N gene than ORF1ab gene, possibly indicating the higher sensitivity of assay for N gene than for the ORF1ab gene. Based on evaluation of either gene, duration of viral shedding appeared to be longer in patients of older age. This finding was similar to the previous studies of Zheng’s study, in which viral load could persist for nearly 30 days in the patients of older than 60 years old [8]. A more prolonged shedding was also seen in female patients and those hospitalized with longer delay, as revealed by survival analysis and Kaplan–Meier curves, which is consistent with the findings from Zhou et al. [17] and Shu et al. [18]. Recent studies have shown that both age and sex had a considerable effect on the outcome of infections and have been associated with underlying differences in immune responses to infection. In the context of COVID-19, both age and sex disparity in disease severity had been displayed, potentially through different immunological mechanisms of disease progression between sex and ages. Takehiro Takahashi’s study had provided robust evidence showing substantial differences in the baseline immune capabilities between male and female patients [19]. Among all the differences, female tend to have more Th2-driven immunity in response to pathogens than male [20]. Shearer et al. have proposed aging can lead to a Th1/Th2 imbalance that older individuals are more likely to have a Th2-type dominance [21]. Th1 (including IL-2 and IFN-γ) have been identified to participate in the virus clearance while Th2 cytokine IL-10 serves as a potent inhibitor of Th1 effectors cells [22]. In the premise of different Th1/Th2 induction, it’s logical to determine that female and the older patients were more likely to have prolonged viral shedding.

On the other hand, although disease severity had been frequently related to viral loads, viral duration and viremia in previous studies [8, 23, 24], we failed to determine significant difference in the timing of viral negativity between patients with different severity in the current study. For one reason, the potential correlation might have been compromised by the robust effect from age on the disease severity. This could also be explained by an insufficient data set, where many non-survivors tended to have few consecutive sampling points available, hindering a further assessment of prolonged viral shedding in related to severe disease. According to numerous studies that have addressed the correlation between viral shedding and COVID-19-associated symptoms, the mean period of SARS-CoV-2 viral shedding were slightly higher in patients with COVID-19-associated symptoms compare to asymptomatic patients (25.0 ± 7.8 vs. 23.4 ± 8.9, P = 0.051), however, the proportion of symptomatic patients with viral shedding > 24 days were significantly higher than asymptomatic group [25]. Consistently, a study of 71 patients showed shorter duration of viral shedding in asymptomatic patients than symptomatic group [26]. Specific clinical symptoms, such as fever, have also been associated with longer periods of viral RNA shedding, with OR (95%CI) of 5.200 (1.190–22.726) in a study performed in China [27]. Here we failed to identify any specific symptoms that could be related to duration of viral shedding, which however, need to be replicated by further large scale analysis.

From the start of the disease onset, there was a difference in viral RNA level between patients who were sick earlier, and the difference was also decreasing with the time since onset of symptoms. Hypotheses to explain this decrease are numerous and one of them is that increasing immunity in the population could contribute to the lower viral loads by reaching a herd immunity threshold as the outbreak progressed. There are also other possibilities that as the outbreak progressed, the awareness of the disease could have increased among the population, with more mild patients seeking medical care at late epidemic, whereas early in the outbreak more severe cases with expected higher viral shedding were tested. This bias although might have been adjusted by multivariate analysis, was ineradicable. Another finding is the correlation between the low COVID-19-specific IgM antibodies and the high initial viral loads observed from the infected patients, indicating the inefficiency of the patients in producing humoral immunity against the virus.

Our study has several limitations. First, the high median Ct value tested at symptom onset when the viral loads was supposed to be very high might be related to detection instruments, assays or operators. Second, no test was performed for asymptomatic cases, thus whether the results will remain same with reference to the asymptomatic group cannot be addressed at this moment. In addition, some of the predesigned sampling was missing, which might cause bias in estimating the viral shedding due to the inequivalent sampling and reduced sample size. Especially for the non-survivors, only sparse points of sampling were performed for tests due to the emergency situation, thus the data of non-survivors is insufficient to be applied in the detailed analysis on the viral kinetics or related factors. Third, although predominant part of the patients received antiviral therapy during their hospitalization, detailed information on the therapy dosage or duration were not collected, therefore how the antiviral treatments can affect the viral replication cannot be evaluated in the current study.

In conclusion, we displayed the temporal viral shedding along the hospitalization duration. The temporal course of viral loads that was simulated by viral dynamics trajectory based on N gene and ORF gene allowed for a prediction of the SARS-CoV-2 virus at different clinical stages. These findings could serve as better guidance for the duration of infection prevention measures.

## Supplementary Information

**Additional file 1: Table 1.** Demographics and clinical characteristics of COVID-19 patients between SVS and LVS groups. **Table 2.** The association between delayed time to SARS-CoV-2 RNA negativity and symptoms on admission for COVID-19 patients. **Table 3.** Outbreak kinetics of SARS-CoV-2 viral loads level over three outbreak stages expressed as N and ORF Ct value for COVID-19 patients. **Figure 1.** IgM and IgG antibody titers compared between patients with high vs. low viral loads based on N gene (A and C) and ORF gene (B and D).

## Data Availability

After publication, the data and materials used in the study will be made available to others upon a proper request from the corresponding author Wei Liu at lwbime@163.com or liuwei@bmi.ac.cn.
