# Supplementary material for: Prolonged viral shedding of SARS-CoV-2 and related factors in symptomatic COVID-19 patients: a prospective study
Source: BMC Infect Dis. 2021 Dec 27;21:1282. doi: 10.1186/s12879-021-07002-w (PMC8711078; doi:10.1186/s12879-021-07002-w)
Supplement: Supplementary file 1 — Additional file 1: Table 1. Demographics and clinical characteristics of COVID-19 patients between SVS and LVS groups. Table 2. The association between delayed time to SARS-CoV-2 RNA negativity and symptoms on admission for COVID-19 patients. Table 3. Outbreak kinetics of SARS-CoV-2 viral loads level over three outbreak stages expressed as N and ORF Ct value for COVID-19 patients. Figure 1. IgM and IgG antibody titers compared between patients with high vs. low viral loads based on N gene (A and C) and ORF gene (B and D). [file 12879_2021_7002_MOESM1_ESM.docx]

**Additional Materials**

Additional Table 1. Demographics and clinical characteristics of COVID-19 patients between SVS and LVS groups.

Additional Table 2. The association between delayed time to SARS-CoV-2 RNA negativity and symptoms on admission for COVID-19 patients.

Additional Table 3. Outbreak kinetics of SARS-CoV-2 viral loads level over three outbreak stages expressed as N and ORF Ct value for COVID-19 patients.

Additional Figure 1. IgM and IgG antibody titers compared between patients with high *vs.* low viral loads based on N gene (A and C) and ORF gene (B and D).

**Additional Table 1. Demographics and clinical characteristics of COVID-19 patients between SVS and LVS groups.**

| **Characteristics** | **N gene** | | | **P value** | **ORF gene** | | | **P value** |
| --- | --- | --- | --- | --- | --- | --- | --- | --- |
|  | **Total**  **(N=205)** | **SVS**  **(n=105)** | **LVS**  **(n=100)** |  | **Total**  **(N=28)** | **SVS**  **(n=14)** | **LVS**  **(n=14)** |  |
| **Age, years (median, IQR)** | 63 (48-70) | 60 (44-69) | 65 (55-70) | 0.057 | 64 (58-70) | 64 (56-68) | 64 (61-74) | 0.408 |
| ≤60 | 87 (42.4) | 53 (50.5) | 34 (34.0) | 0.017 | 8 (28.6) | 6 (42.9) | 2 (14.3) | 0.209 |
| >60 | 118 (57.6) | 52 (49.5) | 66 (66.0) |  | 20 (71.4) | 8 (57.1) | 12 (85.7) |  |
| **Sex, male, n (%)** | 109 (53.2) | 64 (61.0) | 45 (45.0) | 0.022 | 12 (42.9) | 6 (42.9) | 6 (42.9) | 1.000 |
| **Interval^#^, days, median (IQR)** | 9 (7-14) | 8 (5-10) | 12 (9-17) | <0.001 | 9 (7-10) | 8 (6-9) | 11 (8-14) | 0.012 |
| ≤10 | 124 (60.5) | 84 (80.0) | 40 (40.0) | <0.001^*^ | 21 (75.0) | 14 (100.0) | 7 (50.0) | 0.006 |
| >10 | 81 (39.5) | 21 (20.0) | 60 (60.0) |  | 7 (25) | 0 (0) | 7 (50.0) |  |
| **Length of stay, days, median (IQR)** | 24 (21-28) | 23 (19-26) | 24 (23-29) | 0.001 | 22 (20-27) | 20 (18-26) | 23 (21-29) | 0.166 |
| **Clinical manifestation on admission, n (%)** |  |  |  |  |  |  |  |  |
| Fever | 168 (82.0) | 85 (81.0) | 83 (83.0) | 0.703 | 17 (60.7) | 8 (57.1) | 9 (64.3) | 1.000 |
| Cough | 131 (63.9) | 58 (55.2) | 73 (73.0) | 0.008^*^ | 17 (60.7) | 7 (50.0) | 10 (71.4) | 0.440 |
| Fatigue | 53 (25.9) | 25 (23.8) | 28 (28.0) | 0.493 | 7 (25.0) | 3 (21.4) | 4 (28.6) | 1.000 |
| Anhelation | 54 (26.3) | 29 (27.6) | 25 (25.0) | 0.670 | 6 (21.4) | 3 (21.4) | 3 (21.4) | 1.000 |
| Nausea | 16 (7.8) | 10 (9.5) | 6 (6.0) | 0.347 | 3 (10.7) | 2 (14.3) | 1 (7.1) | 1.000 |
| Diarrhea | 30 (14.6) | 19 (18.1) | 11 (11.0) | 0.151 | 2 (7.1) | 2 (14.3) | 0 (0) | 0.481 |
| Anorexia | 29 (14.1) | 13 (12.4) | 16 (16.0) | 0.457 | 3 (10.7) | 3 (21.4) | 0 (0) | 0.222 |
| **Any comorbidity, n (%)** | 88 (42.9) | 44 (41.9) | 44 (44.0) | 0.762 | 11 (39.3) | 5 (35.7) | 6 (42.9) | 1.000 |
| Hypertension | 65 (31.7) | 32 (30.5) | 33 (33.0) | 0.698 | 7 (25.0) | 4 (28.6) | 3 (21.4) | 1.000 |
| Diabetes | 34 (16.6) | 19 (18.1) | 15 (15.0) | 0.551 | 8 (28.6) | 3 (21.4) | 5 (35.7) | 0.678 |
| Cardiovascular disease | 14 (6.8) | 6 (5.7) | 8 (8.0) | 0.517 | 0 (0) | 0 (0) | 0 (0) | 1.000 |
| Cerebral infarction | 5 (2.4) | 3 (2.9) | 2 (2.0) | 1.000 | 1 (3.6) | 0 (0) | 1 (7.1) | 1.000 |
| **Disease severity, severe, n (%)** | 15 (7.3) | 7 (6.7) | 8 (8.0) | 0.714 | 4 (14.3) | 1 (7.1) | 3 (21.4) | 0.596 |

IQR, interquartile range; SVS, short viral shedding; LVS, long viral shedding. N/ORF gene, indicate any positive of N and ORF genes was considered as positive.^#^. Indicates interval between disease onset and hospital admission.

^*^. The difference between SVS and LVS groups was statistically significant after adjustment for age, sex, days from symptom onset to admission and clinical manifestation of cough.

**Additional Table 2. The associations between delayed time to SARS-CoV-2 RNA negativity and symptoms on admission for COVID-19 patients.**

| **Symptoms** | **Crude** | |  | **Adjusted^*^** | |
| --- | --- | --- | --- | --- | --- |
|  | **HR (95% CI)** | **P value** |  | **HR (95% CI)** | **P value** |
| **Fever** |  |  |  |  |  |
| No | Reference |  |  | Reference |  |
| Yes | 1.06 (0.76-1.49) | 0.716 |  | 1.04 (0.74-1.47) | 0.823 |
| **Cough** |  |  |  |  |  |
| No | Reference |  |  | Reference |  |
| Yes | 0.78 (0.59-1.03) | 0.076 |  | 0.78 (0.59-1.04) | 0.086 |
| **Fatigue** |  |  |  |  |  |
| No | Reference |  |  | Reference |  |
| Yes | 1.01 (0.74-1.37) | 0.966 |  | 1.01 (0.74-1.39) | 0.927 |
| **Anhelation** |  |  |  |  |  |
| No | Reference |  |  | Reference |  |
| Yes | 1.21 (0.89-1.64) | 0.225 |  | 1.32 (0.96-1.82) | 0.084 |
| **Nausea** |  |  |  |  |  |
| No | Reference |  |  | Reference |  |
| Yes | 1.52 (0.91-2.53) | 0.108 |  | 1.42 (0.85-2.37) | 0.180 |
| **Diarrhea** |  |  |  |  |  |
| No | Reference |  |  | Reference |  |
| Yes | 1.39 (0.94-2.05) | 0.096 |  | 1.34 (0.9-1.98) | 0.151 |
| **Anorexia** |  |  |  |  |  |
| No | Reference |  |  | Reference |  |
| Yes | 0.97 (0.66-1.44) | 0.893 |  | 0.99 (0.67-1.47) | 0.958 |

HR, hazard ratio; CI, confidence interval. The univariate and multivariate Cox regression model were used in the analysis.

**^*^**. Independent variables including age, sex and any comorbidity were included into multivariate Cox regression model.

**Additional Table 3. Outbreak kinetics of SARS-CoV-2 viral loads level over three outbreak stages expressed as N and ORF Ct value for COVID-19 patients.**

| **Outbreak stages** | **Crude** | |  | **Adjusted^*^** | |
| --- | --- | --- | --- | --- | --- |
|  | **OR (95%CI)** | **P value** |  | **OR (95%CI)** | **P value** |
| **N gene** |  |  |  |  |  |
| 2020.01.01−2020.01.24 | Reference |  |  | Reference |  |
| 2020.01.25−2020.01.30 | 0.94 (0.68-1.29) | 0.685 |  | 0.96 (0.73-1.27) | 0.786 |
| 2020.01.31−2020.03.05 | 0.99 (0.72-1.38) | 0.971 |  | 1.53 (1.07-2.20) | 0.021 |
| **ORF gene** |  |  |  |  |  |
| 2020.01.01−2020.01.24 | Reference |  |  | Reference |  |
| 2020.01.25−2020.01.30 | 1.13 (0.81-1.58) | 0.464 |  | 1.18 (0.88-1.60) | 0.273 |
| 2020.01.31−2020.03.05 | 1.29 (0.91-1.84) | 0.150 |  | 1.94 (1.32-2.87) | 0.001 |

OR, odds ratio; CI, confidence interval. The three outbreak stages were classified to attain comparable case numbers based on the symptom onset date of the patients. Generalized estimation equation (GEE) model was used in the analysis.

**^*^.**The adjusted variables including age, sex, days from symptom onset to admission and disease severity.


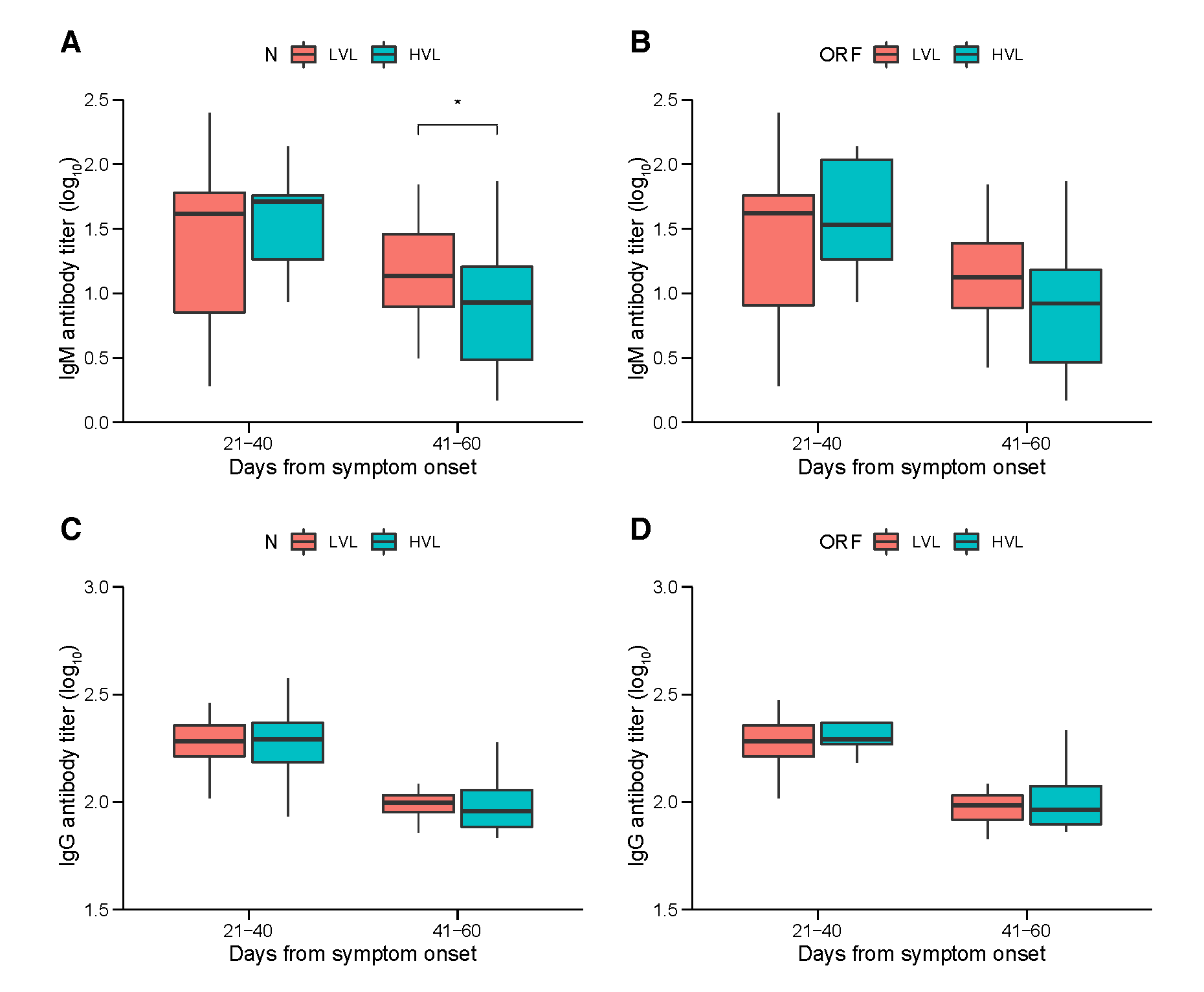


**Additional Figure 1. IgM and IgG antibody titers compared between patients with high *vs.* low viral loads based on N gene (A and C) and ORF gene (B and D).**

The SARS-CoV-2 viral loads was divided into high viral loads (HVL) group and low viral loads (LVL) group based on the mean of the minimum Ct value (herein Ct of 36.3 for N gene and Ct of 37.9 for ORF gene). The SARS-CoV-2-specific IgM and IgG antibodies were measured by using a seroFlash SARS-CoV-2 IgG/IgM ELISA fast kit (Epigentek, USA) with a recombinant SARS-CoV-2 antigen (Spike protein). The antibody titers were compared between HVL and LVL groups based on wilcoxon rank sum test, asterisk represents significant difference (P < 0.05).
